# Efficiency of Four Irrigation Needles in Curved Simulated Root Canals

**DOI:** 10.3390/dj14050278

**Published:** 2026-05-07

**Authors:** Benedicte Elisabeth Strand, Marianne Lægreid, Inge Fristad

**Affiliations:** Department of Clinical Dentistry, Medical Faculty, University of Bergen, 5009 Bergen, Norway; benedicte.e.strand@student.uib.no (B.E.S.); marianne.legreid@uib.no (M.L.)

**Keywords:** irrigation, endodontics, irrigation needles, efficiency, absorbance

## Abstract

**Background/Objectives:** Effective irrigation of the apical third remains one of the greatest challenges in root canal treatment, particularly in curved canals where anatomical complexity restricts irrigant penetration. This in vitro study evaluated the irrigation efficacy of four syringe-needle designs under standardized conditions. **Methods:** Ten transparent resin blocks with approximately 30° curved canals were instrumented to size 30/0.04 taper. Four irrigation needle designs were tested: flat open-ended (27G), notched open-ended (27G), double-vented closed-ended (27G), and finally a flexible polypropylene closed-ended (IrriFlex, 30G) used as a reference. Canals were filled with methylene blue and irrigated dynamically with distilled water. Residual dye was quantified spectrophotometrically at 665–668 nm. Data were analyzed using ANOVA with post hoc testing (*p* < 0.05). **Results:** Significant differences were found among the needle designs. The flexible polypropylene needle showed the lowest absorbance values and performed significantly better than both the flat open-ended and double-vented metallic needles. The notched open-ended needle demonstrated irrigation efficacy comparable to the flexible needle. The double-vented metallic needle exhibited the highest residual dye levels, indicating the poorest irrigation performance. **Conclusions:** Needle design significantly influences irrigation efficacy in curved root canals. Flexible and notched designs enhanced apical dye removal compared with conventional metallic open-ended and side-vented needles. Differences in performance appear to be governed by a combination of vent configuration, needle flexibility, penetration depth, and fluid-dynamic behavior rather than needle gauge alone.

## 1. Introduction

An essential part of root canal treatment (RCT) is the thorough cleaning and shaping of the root canal system, collectively referred to as chemo-mechanical preparation. This process involves removing organic and inorganic debris, as well as infected tissue, through the combined use of mechanical instrumentation and chemical irrigants. Irrigation plays a major role in chemo-mechanical preparation, as instruments are unable to reach all areas of the complex root canal system [1].

Depending on the irrigant used, irrigation serves multiple functions, including lubrication between the instrument and dentin, enhancement of the cutting efficiency of endodontic files, dissolution of organic tissue, and facilitation of debris dislodgement and removal from the canal. In addition, irrigation provides a flushing action and exerts antimicrobial effects. Importantly, irrigation represents the only means of accessing and influencing regions of the root canal system that remain untouched by mechanical instrumentation. Anatomical complexities such as sharp curvatures, lateral canals, narrow isthmuses, fins, and apical deltas further increase the challenges associated with effective root canal treatment.

In recent years, several advanced irrigation systems have been developed, all of which claim to improve irrigation efficiency by delivering the irrigant as close as possible to remote and intricate areas of the root canal system [2]. Passive ultrasonic irrigation, negative pressure systems, sonic devices, and laser-assisted irrigation techniques are among these methods [2,3]. In addition, heating of sodium hypochlorite is a well-documented strategy to enhance tissue dissolution and antimicrobial efficacy [4]. Despite these technological advancements, conventional syringe irrigation remains the predominant clinical method used by both general dental practitioners and endodontic specialists [5,6,7,8].

Effective cleaning of the apical third remains particularly challenging, raising concerns about the efficiency of syringe irrigation performed with relatively rigid stainless-steel needles [9]. Inadequate cleaning in this region may contribute to persistent infection and treatment failure.

Various irrigation needle designs have been developed to improve the effectiveness of syringe irrigation. Depending on whether the irrigant is delivered apically through the tip or laterally via side vents, needles are classified as open-ended or closed-ended [8,10,11]. Open-ended designs include flat-, beveled-, and notched-tip needles, whereas close-ended needles feature single, double or multiple side vents [5,11]. More recently, innovations in both needle design and materials, such as flexible and brush-covered needles, have expanded available options [12].

Needle tip design directly influences flow patterns, velocity and apical pressure, all of which affect irrigation efficacy and safety [5]. Irrigation efficiency is further influenced by root canal anatomy, apical size and curvature, as well as needle choice and flow rate, which together determine irrigant penetration into difficult apical regions [8,13]. Open-ended needles generate a high-velocity apical jet that enhances irrigant penetration and shear stress but increases the risk of apical extrusion [11,13]. In contrast, closed-ended needles direct irrigant laterally, reducing apical pressure and improving safety, though often at the cost of apical penetration [11,13].

Since conventional syringe irrigation remains widely used in clinical practice, it is important to understand the performance of commonly available needle designs. Stainless-steel needles are typically used in 27-gauge, whereas flexible polypropylene needles are commonly manufactured in 30-gauge to allow deeper penetration into curved canals [14]. Given these considerations, it remains unclear how commonly used needle designs differ in their ability to clean curved canals.

In this study, we investigated the irrigation efficacy of four irrigation needle designs to clarify how differences in gauge, material, and vent configuration influence cleaning in curved canals. Three metallic 27-gauge needles were evaluated alongside a flexible 30-gauge reference needle, selected because its size corresponds to the dimensions of the curved canal. By analyzing their ability to remove methylene blue dye from standardized artificial root canals, the aim was to identify which needle design provides the most effective irrigation under controlled conditions.

## 2. Materials and Methods

### 2.1. Specimen Preparation

Ten transparent resin blocks (Shenzhen Flydent Medical Co., Shenzhen, China) with simulated root canals exhibiting approximately 30° curvature were prepared. Initial canal instrumentation was performed using a size #10 K-file (Dentsply Sirona, Charlotte, NC, USA), followed by sequential enlargement up to size #25 with hand files using the balanced-force technique. Final preparation was completed to size #30 using a mechanically driven R-motion file (FKG, Crêt-du-Locle, Switzerland) with a 0.04 taper. All instruments were used under continuous irrigation to full working length, extending to the simulated apical constriction, to create a continuously tapered canal shape.

To facilitate removal of methylene blue between experiments, a small opening at the simulated apical constriction was maintained using a size #10 K-file (Dentsply Sirona, USA). During irrigation experiments, this constriction was sealed with a rubber stopper to prevent apical fluid leakage.

### 2.2. Dye Application

Methylene blue solution (Cerkamed, Stalowa Wola, Poland) was introduced into the pre-dried canal of each resin block using a 30-gauge needle (Huizhou Tongmuyuan Technology, Huizhou City, China) in a back-filling mode to ensure complete coverage to the full working length. To standardize conditions and minimize bias, the same ten resin blocks were used across all irrigation needle groups. Between tests, the rubber seal was removed, and the blocks were thoroughly rinsed with distilled water to ensure complete dye removal. After thorough drying of the resin blocks, rubber seal was reinserted, and the canals were filled with concentrated methylene blue.

### 2.3. Experimental Groups

**Figure 1:**
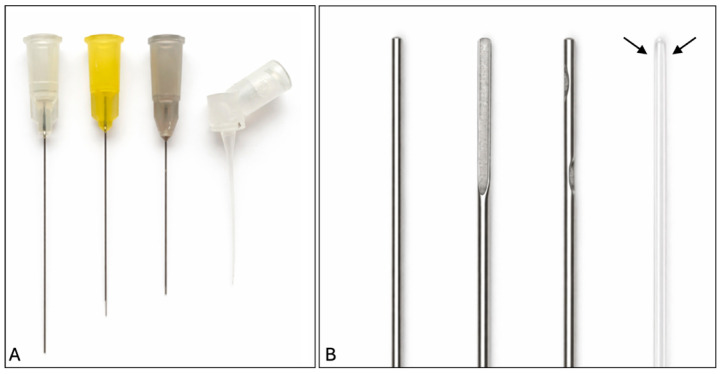
Photo showing the four needle designs used in the study. (**A**) From left to right: flat open-ended, notched open-ended, double-vented closed-ended, and flexible closed-ended. (**B**) Magnified view of the four needles in the same order. The arrows indicate the position of the two vents on the flexible double-vented closed-ended needle.

Four irrigation needle designs were tested (Figure 1). Group 1 included a flat open-ended 27-gauge stainless-steel needle (Huizhou Tongmuyuan Technology, Huizhou City, China); Group 2 a notched open-ended 27-gauge stainless-steel needle (Simplee, Madrid, Spain); Group 3 a double-vented closed-ended 27-gauge stainless-steel needle (Directa, Kiel, Germany); and finally Group 4, the IrriFlex needle, a flexible polypropylene closed-ended 30-gauge needle (Produits Dentaires SA, Vevey, Switzerland). The last needle served as a reference because the size was equivalent to the size of the canal.

### 2.4. Irrigation Procedure

After repeated preoperative training, irrigation was performed by the same operator using a 10 mL syringe (Becton Dickinson S.A., Madrid, Spain) filled with 5 mL of distilled water at a flow rate of 10 mL/min under dynamic irrigation. The procedure included a rubber stopper positioned on the needle at working length. Apical penetration was performed until resistance was encountered (see Section 2.5), followed by repeated up-and-down motions, simulating commonly used clinical procedures. Ten samples of remaining dye per experimental group were collected from the artificial canals using a 10 µL pipette connected to a 30-gauge flat open-ended needle, ensuring inclusion of fluid from the apical region.

### 2.5. Needle Penetration Depth

To control for penetration depth, each needle was inserted in the ten resin blocks until resistance was encountered. Each resin block together with the inserted needle was photographed using a microscope (Eclipse 80i Microscope, Nikon, Tokyo, Japan) connected to Nikon NIS-elements BR imaging software, version 5.x (Nikon Instruments Inc., Tokyo, Japan). The apical distance of the needle tip relative to the working length was measured and expressed as mean with the standard error of the mean.

### 2.6. Spectrophotometric Analysis

Residual methylene blue within the canals was quantified using an absorbance spectrophotometer (Varioskan LUX Microplate Reader, Thermo Fisher Scientific, Waltham, MA, USA). Fluid samples were transferred into wells of a 96-well microplate, requiring a minimum of 100 µL per well for reliable measurement. Because recovered volumes were insufficient, samples were diluted with 90 µL of distilled water prior to analysis.

Positive controls (10 µL methylene blue diluted with 90 µL distilled water) and negative controls (100 µL distilled water) were included. Based on pilot data, undiluted methylene blue exceeded the measurable range of the spectrophotometer. Absorbance was measured at 665–668 nm, the peak absorption range for methylene blue [15]. Final absorbance values were multiplied by 10 to compensate for sample dilution.

### 2.7. Data Analysis

Data for the absorbance values are presented as mean and standard deviation (SD). Because the same resin blocks were reused across experimental groups, absorbance data were analyzed using linear mixed-effects models with resin block included as a random effect and needle type as a fixed effect. Analyses were performed in Stata version 19 (StataCorp, College Station, TX, USA). Post hoc pairwise comparisons were conducted using Scheffé’s method. Model assumptions were evaluated by visual inspection of residual plots and by applying the Shapiro–Wilk test to the model residuals. Statistical significance was set at *p* ≤ 0.05.

The penetration depth data did not fulfil the assumptions for parametric testing. Therefore, non-parametric statistical analyses were performed using the Mann–Whitney U test, based on rank sums. To account for multiple comparisons, Bonferroni correction was applied. Data are presented as median and interquartile range (IQR). Statistical significance was set at *p* ≤ 0.05.

## 3. Results

### 3.1. Needle Penetration Depth

**Figure 2:**
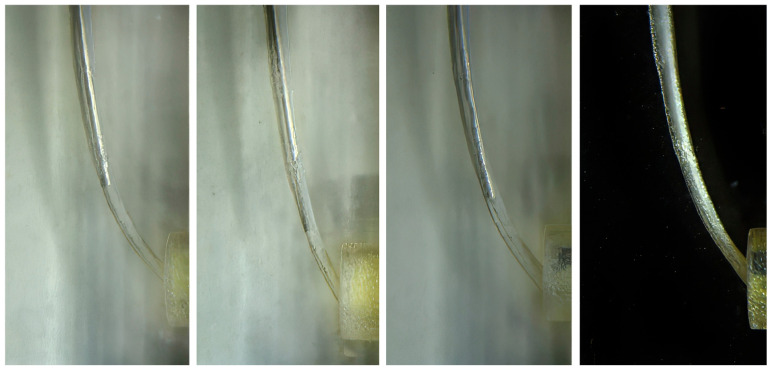
Images of needle penetration depths. From **left** to **right**: flat open-ended, notched open-ended, double-vented closed-ended, and flexible closed-ended.

**Figure 3:**
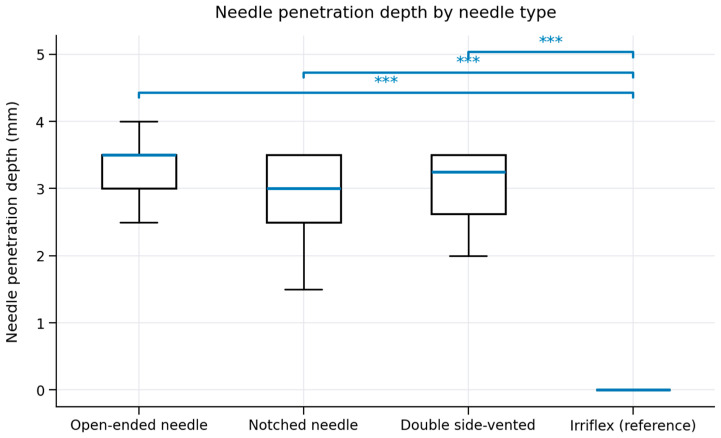
Boxplots showing needle penetration depth for the different irrigation needles. The boxes represent the interquartile range with the median indicated by the horizontal line; whiskers denote minimum and maximum values. Statistical significance versus the Irriflex reference was assessed using the Mann–Whitney U test with Bonferroni correction (*** *p* ≤ 0.001).

Needle penetration depth findings are shown in Figure 2, and a summary of these measurements is presented in Figure 3. Small variations were observed among the 27-gauge metallic needles, whereas the flexible polypropylene needle consistently reached the working length. Penetration and morphometric analyses indicated that the notched needle created a small apical space between the needle tip and the canal wall, even without deeper penetration. In contrast, the flat open-ended and the side-vented metallic needles may have restricted reflux of the irrigation solution, thereby limiting apical fluid exchange. Furthermore, the more coronally position of the side-vented openings further reduced the effective apical penetration depth.

### 3.2. Residual Dye Visualization

**Figure 4:**
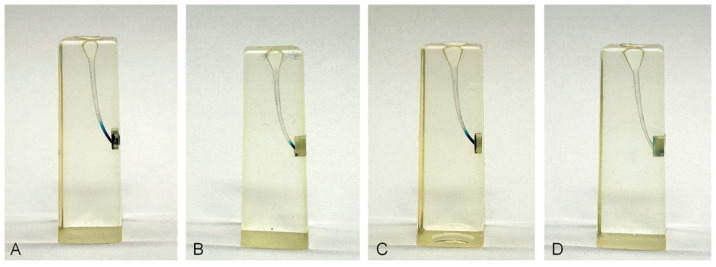
Resin blocks after irrigation with 5 mL distilled water for 30 s. (**A**) Flat open-ended needle, (**B**) notched open-ended needle, (**C**) double-vented closed-ended needle, and (**D**) flexible closed-ended needle. Differences between the needle groups are most apparent in the apical portion of the root canals.

After irrigation of the simulated root canals, a visual evaluation was performed before the collected fluid was transferred to the 96-well microplate and diluted with distilled water. A representative resin block from each experimental group is shown after irrigation (Figure 4). Residual methylene blue in the apical portion of the canal illustrates the irrigation efficacy of each needle design. A greater amount of remaining dye indicates less effective irrigation.

**Figure 5:**
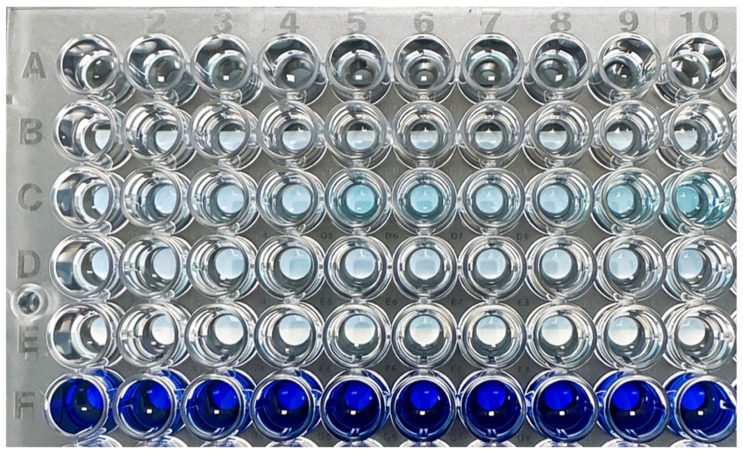
A 96-well microplate showing the distribution of all samples. The four experimental groups were allocated to rows A–D, with columns 1–10 representing individual samples from each group. The negative and positive control groups are shown in rows E and F, respectively.

Figure 5 shows the 96-well microplate with the diluted samples from the test groups. The color intensity represents the amount of methylene blue remaining in the simulated canals.

### 3.3. Spectrophotometric Absorbance Measurements

**Figure 6:**
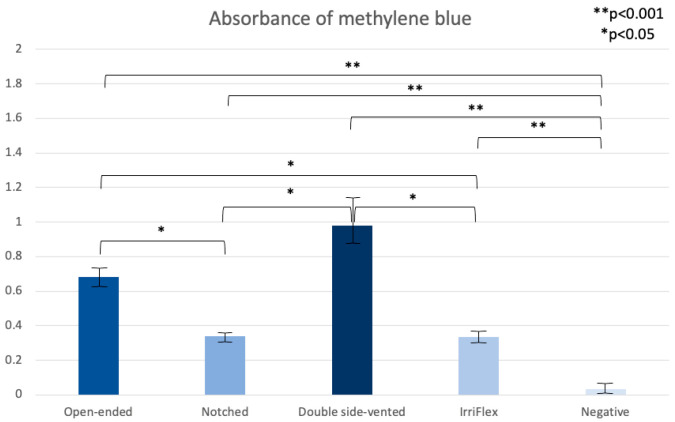
Absorbance of methylene blue after irrigation using different needle designs. Bars represent mean values with standard deviation for each test group. Statistically significant differences between groups are indicated (* *p* < 0.05; ** *p* < 0.001).

Spectrophotometric absorbance measurements of methylene blue revealed significant differences in irrigation efficacy among the tested needle designs (Figure 6). Lower absorbance values indicate more effective removal of methylene blue from the simulated root canals, whereas higher absorbance values reflect greater residual dye, and thus reduced irrigation efficacy. The double-vented closed-ended needle demonstrated the highest mean absorbance value, indicating the poorest irrigation performance. The flat open-ended needle also exhibited relatively high absorbance values, indicating lower irrigation efficacy compared with the remaining needle designs.

In contrast, both the notched needle and the flexible polypropylene needle demonstrated significantly lower absorbance values, indicating superior irrigation performance. The flexible polypropylene needle group exhibited the lowest mean absorbance levels and differed significantly from both the metallic double-vented closed-ended and the flat open-ended needle groups (*p* < 0.05). Similar results were observed for the notched needle. The negative control group exhibited minimal absorbance values, confirming the validity of the spectrophotometric measurements and serving as a baseline reference.

The positive control showed high absorbance values (mean ≈ 41), representing maximal methylene blue retention. Because this value was far outside the dynamic range of the experimental groups, it was omitted from Figure 6 and from comparative statistical analysis. Its inclusion would have prevented meaningful visualization of differences among the test groups. Nevertheless, its markedly elevated absorbance verified the sensitivity and validity of the assay.

## 4. Discussion

The favorable performance of the flexible polypropylene needle is consistent with previous studies demonstrating superior performance of flexible needle designs [14,16,17]. However, in contrast to earlier reports, the notched metallic needle also demonstrated favorable performance, with irrigation efficacy comparable to that of the polypropylene needle [11]. It should be noted that the present results are based on curved root canals, an anatomical feature that inherently complicates effective irrigation of the apical region [18]. The intention was also to evaluate the performance of commercially available 27-gauge metallic needles, which are widely used in clinical practice. For this reason, the flexible needle was used as a reference, as it adapted well to the artificial canal.

### 4.1. Fluid Dynamics

Irrigation performance in the root canal is largely governed by fluid dynamics, which are influenced by how the irrigation solution is delivered into the canal system [19]. Different needle designs generate distinct flow patterns that influence irrigant penetration, wall shear stress and debris removal. Wall shear stress is created by velocity gradients within the fluid and plays a crucial role in mechanical detachment of debris, biofilm or dye [20]. This shear stress is highest near the needle outlet and decreases toward the canal walls due to viscosity-dependent frictional forces [21]. Open-ended needles generate a direct apical jet, producing high shear stress near the apex but also increasing apical pressure and the risk of irrigant extrusion. Closed-ended side-vented designs distribute the irrigant laterally, reducing apical pressure and improving safety but often limiting irrigant penetration into the apical region [11].

Despite sharing a closed-ended double-vented configuration, the flexible and metallic side-vented needles performed differently. The flexible needle’s vents are positioned immediately adjacent to the tip, enhancing apical delivery. In contrast, the metallic needle places its vents more coronally, diminishing apical flow intensity and reducing irrigant replacement at working length. While both designs promote shear stress along canal walls, effective irrigation also requires robust coronally directed flow to transport detached debris out of the canal. In line with previous studies, vent position, needle flexibility and ability to track canal curvature, collectively explain the improved performance observed with the flexible polypropylene needle [22].

In addition to needle design and flow rate, shear stress is influenced by multiple factors, including apical diameter, canal taper, needle penetration depth, irrigation technique and needle size [19,23]. Needle design therefore interacts with—rather than fully determines—overall irrigation efficacy.

### 4.2. Flow Rate Considerations

Flow rate is determined by the force applied to the syringe plunger and the resulting velocity of irrigant exiting the needle. The irrigant flow rate directly influences apical pressure, a critical determinant of both irrigation efficacy and safety [24]. Although irrigant volume and delivery time were standardized, the true flow rate inevitably varies with needle design, gauge, and canal morphology, thereby influencing irrigation potential. Curved canals restrict irrigant movement, making a consistent flow rate difficult to maintain, particularly in the apical third. The absence of a mechanical syringe-depression device also introduced natural variation in plunger force between samples. Although this variability represents a potential source of bias, it reflects clinical practice, where identical plunger force cannot be ensured. Some degree of flow-rate variation between samples is therefore unavoidable. As demonstrated in this study, flow rate and fluid dynamics may be compromised in clinical practice when there is a mismatch between needle size and canal dimensions, particularly in curved root canals.

### 4.3. Dynamic Irrigation

Manual dynamic activation (MDA) is known to enhance irrigant exchange compared with static irrigation, even when the apical diameter remains relatively small [25]. This is largely achieved by the needle motion, which promotes turbulence and improves fluid turnover. Static irrigation depends solely on the depth of needle penetration, whereas dynamic irrigation is influenced by better irrigant exchange. This exchange is affected by canal taper and diameter and is therefore indirectly related to needle penetration depth [26]. In the present study, up-and-down needle motions were used in all samples to stimulate common clinical practice. MDA is widely used by both general dental practitioners and endodontic specialists, strengthening the clinical relevance of this study and its simulation of conventional irrigation procedures. While simulated root canals are widely used in irrigation research, they do not accurately reproduce the structural and fluid-dynamic complexity of natural teeth. These shortcomings are particularly relevant when biofilm formation and microbiological interactions with the irrigant are studied [27]. Turbulent flow generated through MDA enhances mixing, improves debris suspension, and increases the likelihood of irrigant reaching complex canal regions [19]. Although numerous device-assisted activation techniques exist [28], clinical outcomes remain inconsistent due to methodological variability. Nevertheless, there is consensus that irrigant agitation, regardless of the activation technique, improves irrigant distribution and effectiveness, making activation at the end of the canal preparation more effective than static syringe irrigation alone. MDA remains a widely used, cost-effective and clinically relevant method for improving irrigant distribution, further strengthening the clinical relevance of the present findings.

### 4.4. Apical Dimension, Taper and Curved Canals

Apical preparation size and taper strongly influence irrigant penetration and efficacy, particularly in curved canals. Previous studies indicate that curvatures greater than 24° increase the need for larger apical size for effective irrigation [18]. In this study, curved canals were prepared to a size 30 with a 0.04 taper, dimensions consistent with routine clinical practice in curved root canals.

Larger apical preparations increase canal volume and provide greater space for irrigant flow and debris removal, potentially improving bacterial elimination [29]. Although larger apical sizes can improve irrigant flow and debris removal, they also carry an inherent risk of procedural complications such as transportation, ledging or perforation [18]. Consequently, further apical enlargement in curved root canals is not always practical or desirable because of the increased risk of root perforation, fracture, or structural weakening [18].

In the present study, a 0.04 taper was used, although a greater taper can improve debris removal by increasing canal volume and enhancing irrigant penetration [30]. However, only a very large taper (0.10) appears to equalize debris removal in smaller apical preparations (e.g., size 20), when compared with larger apical preparations (e.g., size 40) [30]. Clinically, the choice is between increasing the taper, reducing needle size, or ultimately combining both approaches.

### 4.5. Needle Penetration Depth

Needle penetration depth is influenced by canal curvature, needle rigidity and needle diameter. Rigid metallic needles generally cannot follow severe curvature and therefore remain short of working length, which may reduce irrigant exchange in the apical third [19,29,31]. In addition, root canal size, needle design, and needle gauge influence apical penetration in curved canals, resulting in variations in apical pressure generation and apical irrigation efficacy [13].

Flexible polymer-based needles adapt more readily to curved root canals, enabling deeper penetration and improved irrigant delivery. This characteristic likely contributed to the superior performance of the flexible needle in this study [14,25]. However, deeper penetration can also increase apical pressure and the risk of periapical extrusion [11].

Preparation size and needle insertion depth are closely interrelated and must be carefully optimized to ensure effective disinfection while minimizing the risk of extrusion. The combination of a relatively small apical preparation size and the use of a flexible needle created conditions that were particularly favorable among the four needle designs. Conversely, the rigid metallic 27-gauge needles were unable to achieve adequate penetration in the curved canals, likely obstructing irrigant reflux and limiting apical exchange. This may explain the differences among the metallic needle designs, as the notch could have permitted both outward flow and reflux of the irrigant.

### 4.6. Needle Size

Needle gauge influences irrigant flow dynamics, penetration depth, and the ability to reach the apical region. In the present study, 27-guage metallic needles were used for the three stainless-steel designs, whereas the flexible needle was 30-gauge. Smaller-gauge needles typically generate higher flow velocity and allow deeper penetration of the irrigant into narrow canal spaces [25,32,33]. Although reaching short of the apex, the notched 27-gauge metallic needle performed comparably to the 30-gauge flexible needle, demonstrating that needle design may outweigh gauge alone in determining irrigant flow characteristics [11,34]. At the same time, this finding contrasts earlier reports suggesting that smaller-gauge metallic needles were the decisive factor for improved performance [33]. An explanation for the performance of the notched may be a result of the limited obstruction due to the configuration in the notched area allowing both outward and reflux movement of the irrigant.

Although differences in gauge introduce a potential confounding factor, significant differences among the 27-gauge needles themselves indicate that gauge alone cannot explain the observed results. Nonetheless, needle gauge should be considered when interpreting efficacy outcomes, as it inevitably influences irrigant flow and penetration.

## 5. Conclusions

Within the limitations of this in vitro study, needle design significantly influenced irrigation efficacy in curved root canals. The flexible polypropylene needle showed the highest dye-removal capacity and outperformed the open-ended and double-vented metallic designs. The notched metallic needle performed comparably to the flexible needle, underscoring the importance of specific design features such as vent configuration and flexibility.

Multiple factors—including flow rate, shear stress, canal anatomy, apical dimension, taper, penetration depth, gauge, and dynamic activation—collectively contributed to the overall irrigation outcomes. Because of the intricate canal anatomy, restricted irrigant access and extrusion risk, cleaning the apical third continues to be one of the most difficult tasks in endodontic therapy. Future studies should employ controlled flow conditions and investigate the combined effect of the multiple contributing variables to clarify how they interact and to optimize safe and effective irrigation in curved canals.

## Data Availability

The raw data supporting the conclusions of this article will be made available by the authors on request.
